# Eighteen-Month Outcomes Among Pregnant and Nonpregnant Reproductive-Aged People Hospitalized for Coronavirus Disease 2019

**DOI:** 10.1093/ofid/ofae278

**Published:** 2024-07-08

**Authors:** Lisa M Bebell, Ann E Woolley, Kaitlyn E James, Andy Kim, Mary-Ruth Joyc, Kathryn J Gray, Caitlin Radford, Ingrid V Bassett, Adeline A Boatin, Andrea L Ciaranello, Sigal Yawetz, Andrea G Edlow, Ilona T Goldfarb, Khady Diouf

**Affiliations:** Division of Infectious Disease, Department of Medicine, Massachusetts General Hospital, Harvard Medical School, Boston, Massachusetts, USA; Department of Medicine, Division of Infectious Disease, Brigham and Women's Hospital, Harvard Medical School, Boston, Massachusetts, USA; Department of Obstetrics and Gynecology, Massachusetts General Hospital, Harvard Medical School, Boston, Massachusetts, USA; Department of Medicine, Division of Infectious Disease, Brigham and Women's Hospital, Harvard Medical School, Boston, Massachusetts, USA; Department of Medicine, Division of Infectious Disease, Brigham and Women's Hospital, Harvard Medical School, Boston, Massachusetts, USA; Department of Obstetrics and Gynecology, University of Washington School of Medicine, Seattle, Washington, USA; Department of Medicine, Division of Infectious Disease, Brigham and Women's Hospital, Harvard Medical School, Boston, Massachusetts, USA; Division of Infectious Disease, Department of Medicine, Massachusetts General Hospital, Harvard Medical School, Boston, Massachusetts, USA; Department of Obstetrics and Gynecology, Massachusetts General Hospital, Harvard Medical School, Boston, Massachusetts, USA; Division of Infectious Disease, Department of Medicine, Massachusetts General Hospital, Harvard Medical School, Boston, Massachusetts, USA; Department of Medicine, Division of Infectious Disease, Brigham and Women's Hospital, Harvard Medical School, Boston, Massachusetts, USA; Department of Obstetrics and Gynecology, Massachusetts General Hospital, Harvard Medical School, Boston, Massachusetts, USA; Vincent Center for Reproductive Biology, Massachusetts General Hospital, Boston, Massachusetts, USA; Department of Obstetrics and Gynecology, Massachusetts General Hospital, Harvard Medical School, Boston, Massachusetts, USA; Department of Obstetrics and Gynecology, Brigham and Women's Hospital, Harvard Medical School, Boston, Massachusetts, USA

**Keywords:** birth, COVID-19, maternal, pregnancy, SARS-CoV-2

## Abstract

**Background:**

Physiologic and immunologic adaptations in pregnancy may increase the risk of adverse outcomes from respiratory viral infections. However, data are limited on longer-term outcomes after severe acute respiratory syndrome coronavirus 2 (SARS-CoV-2) infection in pregnancy prior to widespread vaccine availability.

**Methods:**

Using electronic health record data, we retrospectively compared 6-, 12-, and 18-month outcomes including death and rehospitalization between pregnant and nonpregnant reproductive-aged individuals hospitalized for SARS-CoV-2 infection between 2020 and 2021 at 2 academic referral hospitals.

**Results:**

There were 190 nonpregnant and 70 pregnant participants. Mean age was 31 years for pregnant and 34 years for nonpregnant participants. For pregnant patients, mean gestational age at coronavirus disease 2019 (COVID-19) diagnosis was 36 weeks, 54% delivered by cesarean, and 97% delivered a live birth. Compared to pregnant participants, nonpregnant participants had a higher prevalence of baseline comorbidities and a higher proportion received mechanical ventilation (84% vs 55%). Index hospitalization complications (31% vs 17%) and mortality (3% vs 0%) were more common in nonpregnant participants. Over 18 months following index hospitalization, 39 (21%) nonpregnant and 5 (7%) pregnant participants were readmitted, most for infection (28/44 [64%]). Most readmissions occurred within 6 months. There were no posthospitalization deaths in the pregnant group.

**Conclusions:**

Pregnant people with severe COVID-19 disease had a low rate of severe adverse outcomes after index hospitalization. The low readmission rate is reassuring that pregnant individuals may not be at higher risk for long-term severe adverse health outcomes after COVID-19 compared to the nonpregnant reproductive-aged population, possibly because any increased risk conferred by pregnancy resolves soon after delivery.

Pulmonary and immunologic adaptations in pregnancy may increase the risk of adverse outcomes from respiratory viral infections [1–3]. However, there are limited data on longer-term outcomes after severe acute respiratory syndrome coronavirus 2 (SARS-CoV-2) infection in pregnancy. Published studies concerning SARS-CoV-2 and other infections in pregnancy may be biased by lower clinical thresholds to treat pregnant individuals conservatively and aggressively. This may lead to earlier hospitalizations, transfer to the intensive care unit (ICU), intubation and mechanical ventilation, and other treatment modalities, rendering study results from nonpregnant populations less applicable to pregnant individuals. Thus, risk of complications from severe coronavirus disease 2019 (COVID-19) disease in pregnancy and outcomes after infection remain poorly defined. Furthermore, few published studies to date have described long-term maternal outcomes after COVID-19 or compared outcomes to otherwise similar nonpregnant individuals.

We compared 6-, 12-, and 18-month outcomes including death and rehospitalization between pregnant and nonpregnant reproductive-aged gender-matched individuals hospitalized for SARS-CoV-2 infection, accounting for demographics, comorbidities, and vital signs and laboratory abnormalities present during index hospitalization at 2 academic tertiary referral hospitals in the Boston area to inform a greater understanding of long-term outcomes after severe COVID-19 disease in the pregnant population.

## METHODS

### Study Population and Recruitment

This is a retrospective descriptive cohort study. Participants were residents of the greater Boston area admitted to Massachusetts General Hospital or Brigham and Women's Hospital between March 2020 and February 2021 prior to the widespread availability of the COVID-19 vaccines and when the Alpha variant of COVID-19 was circulating. For this study, we included pregnant and nonpregnant adults who self-identified as women, were aged 18–50 years, and were hospitalized with COVID-19 infection diagnosed by a positive SARS-CoV-2 quantitative reverse-transcription polymerase chain reaction (qRT-PCR) result. If test results were not available at the time of enrollment, patients were included as having presumed COVID-19 disease based on clinician assessment, with final determination of infection based on SARS-CoV-2 qRT-PCR results. We excluded pregnant people hospitalized for delivery in whom SARS-CoV-2 testing was incidentally found to be positive.

### Data Collection

For the study cohort, baseline demographics, clinical characteristics, laboratory measurements, radiographic studies, and mortality were obtained from the electronic health record (EHR) (Epic Systems, Verona, Wisconsin) by a combination of automated queries using the National Death Index, Research Patient Data Registry, and confirmation through physician medical record review and were entered into a REDCap database. No data on prior SARS-CoV-2 infection or immunity were collected. Hospital readmissions and 6-, 12-, and 18-month follow-up data and outcomes, including loss to follow-up, were assessed by examining the EHR of the Mass General Brigham healthcare system.

### Definitions

Preexisting diabetes was clinically defined as taking glucose-lowering therapy, chart diagnosis of diabetes, or hemoglobin A1C level >6.4%. History of cardiovascular disease was defined as cardiomyopathy, congestive heart failure, coronary artery disease, myocardial infarction, or dyslipidemia prior to pregnancy. Preexisting pulmonary disease was defined as a pre-pregnancy history of bronchiectasis, cystic fibrosis, use of supplementary oxygen or noninvasive ventilation at home/residence, emphysema, interstitial lung disease, asthma, or chronic obstructive pulmonary disease. Our primary exposure was hospitalization for SARS-CoV-2 infection and our primary outcome was readmission within 18 months after the index hospitalization. Our secondary outcome was death within 18 months after the index hospitalization. We excluded follow-up hospitalization for delivery as an outcome.

### Analysis

Participants were defined as having severe COVID-19 in pregnancy if they were pregnant and hospitalized for COVID-19. Participants testing positive for SARS-CoV-2 who were asymptomatic, including those who incidentally tested positive on screening tests during the delivery admission, were excluded. Hospital readmission was defined as a nonelective readmission to the Mass General Brigham system within 18 months of index COVID-19 hospitalization. Index admission and readmission diagnoses were defined by *International Classification of Diseases, Tenth Revision* (*ICD-10*) codes. As this is primarily a descriptive study, we did not use statistical models to assess the relationship between the exposure and outcome. We performed all analyses in R version 4.0.2 (R Project for Statistical Computing), including a Kaplan-Meier curve representing time to readmission in both groups, using the R survival package.

## RESULTS

A total of 190 nonpregnant and 70 pregnant people were included (Table 1). Mean age of pregnant participants was 31 (interquartile range [IQR], 26–35) years for pregnant participants and 34 (IQR, 30–40) years for nonpregnant participants. Distribution of self-reported primary language was similar between groups, and the proportion of individuals living in disadvantaged neighborhood communities, measured by the Area Deprivation Index [4] as a marker for socioeconomic status by census block group, was similar across both groups. However, more pregnant than nonpregnant participants self-reported Black non-Hispanic (27% vs 19%) or Hispanic or Latinx (50% vs 41%) race/ethnicity (Table 1).

**Table 1.** Participant Demographics by Pregnancy Status at Index Admission

Characteristic	Nonpregnant (n = 190)	Pregnant (n = 70)
Age, y, median (IQR)	34 (30–40)	31 (26–35)
Self-reported race/ethnicity, No. (%)		
White non-Hispanic	53 (28)	8 (11)
Black non-Hispanic	37 (19)	19 (27)
Asian non-Hispanic	7 (4)	2 (3)
Hispanic or Latinx	78 (41)	35 (50)
Other/Unknown	15 (8)	6 (9)
Self-reported primary language, No. (%)		
English	124 (67)	43 (61)
Spanish	55 (30)	23 (33)
Portuguese	2 (1)	2 (3)
Haitian Creole	1 (1)	1 (1)
Other	4 (2)	1 (1)
ADI quintile, No. (%)		
1	14 (7)	6 (9)
2	53 (28)	20 (29)
3	55 (29)	21 (30)
4	39 (21)	11 (16)
5	24 (13)	9 (13)

Abbreviations: ADI, Area Deprivation Index; IQR, interquartile range.

Among pregnant participants, 29% were primigravid and 46% were primiparous during the index hospitalization (Table 2). The mean gestational age at COVID-19 diagnosis was 36 (IQR 28–38) weeks. Overall, 54% delivered by cesarean, 97% delivered a live birth, 28% delivered preterm, and 24% were diagnosed with preeclampsia (Table 2). Pregnancy and birth outcome data were not available via EHR review for 2 of 70 (3%). Compared to pregnant participants, nonpregnant participants had a higher prevalence of preexisting risk factors for severe COVID-19, including preexisting pulmonary disease (34% vs 23%), diabetes mellitus (17% vs 6%), history of hypertension (18% vs 10%), history of cardiovascular disease (14% vs 3%), and immunocompromise (16% vs 7%) (Table 3). Prevalence of obesity (determined by pre-pregnancy body mass index [BMI] for pregnant individuals) was similar between groups (57% vs 60%). Vital signs and laboratory parameters were similar between the 2 groups, though nonpregnant participants had a higher prevalence of tachypnea (24% vs 13%) and hypoxemia (22% vs 11%) than pregnant participants. Nonpregnant participants also had a higher prevalence of elevated lactate dehydrogenase (54% vs 27%) and ferritin (21% vs 0%) than pregnant participants (Table 3).

**Table 2.** Characteristics of the Pregnant Population

Characteristic	Pregnant (n = 70^a^)
Gravidity at COVID-19 diagnosis, No. (%)	
1	20 (29)
2	17 (24)
3	18 (26)
4	7 (10)
≥5	8 (11)
Parity at COVID-19 diagnosis, No. (%)	
0	32 (46)
1	21 (30)
2	11 (16)
3	4 (6)
4	2 (3)
Gestational age at COVID-19 diagnosis, y, median (IQR)	36 (28–38)
Pre-pregnancy BMI, kg/m^2^, median (IQR)	30 (26–34)
Live birth, No. (%)	68 (97)
Gestational age at delivery, y, median (IQR)	38 (36–39)
Preterm (<37 wk)	19/68 (28)
Delivery mode, No. (%)	
Vaginal	31/68 (46)
Cesarean delivery	37/68 (54)
Preeclampsia	16/68 (24)

Abbreviations: BMI, body mass index; COVID-19, coronavirus disease 2019; IQR, interquartile range.

^a^Pregnancy outcome data were available for 68 participants.

**Table 3.** Presence of Risk Factors and Biomarkers for Severe Coronavirus Disease 2019 by Pregnancy Status Among Admitted Participants

Characteristic	Nonpregnant (n = 190)	Pregnant (n = 70)
Pre-pregnancy conditions, No. (%)		
Preexisting pulmonary disease	65 (34)	16 (23)
Chronic kidney disease	5 (3)	2 (3)
Diabetes	33 (17)	4 (6)
History of hypertension	35 (18)	7 (10)
History of cardiovascular disease	26 (14)	2 (3)
Obesity (BMI >30 kg/m^2^)	108 (57)	42 (60)
Immunocompromise^a^	31 (16)	5 (7)
Vital signs (hospital admission), No. (%)^b^		
Respiratory rate >24 breaths/min	45 (24)	9 (13)
Heart rate >125 beats/min	20 (11)	8 (11)
SpO_2_ <94% on ambient air	42 (22)	8 (11)
Laboratory tests (hospital admission), No. (%)^c^		
D-dimer >1000 ng/mL	63 (33)	26 (37)
CPK >2× ULN	17 (9)	2 (3)
CRP >100 mg/L	37 (19)	12 (17)
LDH >245 U/L	102 (54)	19 (27)
High-sensitivity cardiac troponin T ≥14 ng/L	22 (12)	3 (4)
Absolute lymphocyte count <0.8	29 (15)	9 (13)
Ferritin >500 μg/L	39 (21)	0 (0)

Abbreviations: BMI, body mass index; CPK, creatine phosphokinase; CRP, C-reactive protein; LDH, lactate dehydrogenase; SpO_2_, oxygen saturation; ULN, upper limit of normal.

^a^Immunocompromise includes history of malignancy, solid organ transplant, bone marrow transplant, and use of immunosuppressive medications.

^b^Respiratory rate missing for 11 participants (2 pregnant), heart rate missing for 6 participants (3 pregnant), and SpO_2_ missing for 8 participants (2 pregnant).

^c^D-dimer missing for 49 participants (29 pregnant), CPK missing for 51 participants (30 pregnant), CRP missing for 68 participants (33 pregnant), LDH missing for 48 participants (25 pregnant), troponin missing for 56 participants (32 pregnant), absolute lymphocyte count missing for 36 participants (24 pregnant), and ferritin missing for 48 participants (30 pregnant).

Index hospitalization length of stay was a median of 5 (IQR, 3–8) days for nonpregnant and 4 (IQR, 3–7) days for pregnant participants (Table 4). The highest level of supplemental oxygen used was similar for both groups (3 L/minute for nonpregnant vs 2 L/minute for pregnant [IQR 2–4 L/min for both]), but a higher proportion of nonpregnant participants received mechanical ventilation (84% vs 55%) and prone position mechanical ventilation (84% vs 55%) and were mechanically ventilated for a longer duration (13 [IQR, 6–19] days vs 8 [IQR, 5–12] days). No pregnant participants were treated with extracorporeal membrane oxygenation (ECMO), while 3 (8%) nonpregnant participants were treated with ECMO. Vasopressor use was similar in nonpregnant compared to pregnant individuals (57% vs 67%, respectively; Table 4).

**Table 4.** Coronavirus Disease 2019 Characteristics and Hospitalization Course by Pregnancy Status Among Admitted Participants

Characteristic	Nonpregnant (n = 190)	Pregnant (n = 70)
Highest level of supplemental O_2_, L/min, median (IQR)	2 (2–4)	3 (2–4)
ICU admission	38 (20)	11 (16)
Proned	20 (53)	4 (36)
Mechanical ventilation	32 (84)	6 (55)
Length of mechanical ventilation, d, median (IQR)	13 (6–19)	8 (5–12)
ECMO	3 (8)	0 (0)
Vasopressors	26 (57)	8 (67)
Length of hospitalization, d, median (IQR)	5 (3–8)	4 (3–7)
Length of ICU admission, d, median (IQR)	9 (3–17)	6 (2–12)
Symptom onset to admission, d, median (IQR)	8 (4–11)	8 (6–10)
Symptom onset to ICU admission, d, median (IQR)	8 (3–11)	7 (3–10)
Complications	58 (31)	12 (17)
Bacterial pneumonia	22 (12)	6 (9)
ARDS	22 (12)	5 (7)
Pneumothorax	1 (1)	1 (1)
Seizure	3 (2)	0 (0)
New-onset myocarditis/pericarditis	4 (2)	0 (0)
Cardiac arrhythmia	10 (5)	1 (1)
Cardiac arrest	1 (1)	0 (0)
Stroke/DVT/PE	8 (4)	0 (0)
Bacteremia/candidemia	4 (2)	1 (1)
Coagulation disorder	4 (2)	1 (1)
Acute renal injury/renal failure	8 (4)	1 (1)
Liver dysfunction (>3×)	23 (12)	7 (10)
Death	6 (3)	0 (0)
In-hospital death	2 (1)	0 (0)
Postdischarge death	4 (2)	0 (0)

Data are presented as No. (%) unless otherwise indicated.

Abbreviations: ARDS, acute respiratory distress syndrome; DVT, deep venous thrombosis; ECMO, extracorporeal membrane oxygenation; ICU, intensive care unit; IQR, interquartile range; O_2_, oxygen; PE, pulmonary embolism.

Index hospitalization outcomes differed between groups, with a higher prevalence of complications in the nonpregnant than pregnant population (31% vs 17%). The complications in both groups were comprised of bacterial pneumonia (12% in the nonpregnant group vs 9% in the pregnant group), acute respiratory distress syndrome (ARDS; 12% vs 7%), liver dysfunction (12% vs 10%), sepsis (5% vs 1%), and cardiac arrhythmia (5% vs 1%). Mortality was 3% in nonpregnant and 0% in pregnant participants (Table 4), with the majority (4 of 6 deaths) occurring postdischarge.

**Figure 1.**
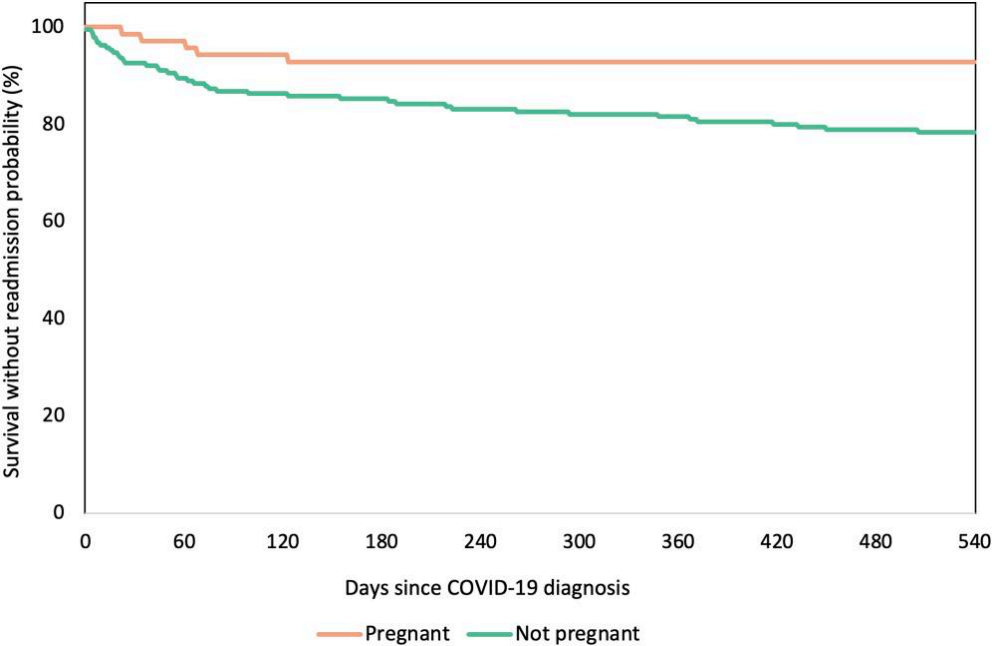
Relationship between pregnancy status and mortality/readmission. Kaplan-Meier curves for the composite outcome of all-cause mortality and readmission are shown, stratified by pregnancy status of patients. Abbreviation: COVID-19, coronavirus disease 2019.

Over the first 18 months following index hospitalization, 39 (21%) nonpregnant and 5 (7%) pregnant participants were readmitted to one of the Mass General Brigham hospitals (Table 5, Figure 1). Nearly two-thirds of readmissions were related to an infectious diagnosis (28/44 [64%]), with gastrointestinal illness the next most common diagnosis (11/44 [25%]). Over the 18-month follow-up period, there were a total of 4 deaths, all in the nonpregnant group (2%). Most readmissions and deaths occurred within the first 6 months after index hospitalization, comprising 26 of 40 (65%) events in the nonpregnant group and 5 of 5 (100%) events in the pregnant group (Table 5).

**Table 5.** Posthospitalization Outcomes of Coronavirus Disease 2019 by Pregnancy Status Among Admitted Reproductive-Aged Participants (n = 190 Nonpregnant, n = 70 Pregnant)

Characteristic	Months After Index Hospitalization						Cumulative Total	
	0–6 mo		6–12 mo		12–18 mo		0–18 mo	
	Non-pregnant	Pregnant	Non-pregnant	Pregnant	Non-pregnant	Pregnant	Non-pregnant	Pregnant
Readmission	25 (13)	5 (7)	7 (4)	0 (0)	7 (4)	0 (0)	39 (21)	5 (7)
Reason for readmission^a^								
Cardiac	5 (3)	2 (3)	…	…	…	…	5 (3)	2 (3)
Thrombotic event	1 (1)	…	…	…	1 (1)	…	2 (1)	…
Pulmonary	2 (1)	…	2 (1)	…	1 (1)	…	5 (3)	…
Infectious	16 (8)	1 (1)	6 (3)	…	5 (3)	…	27 (14)	1 (1)
Gastrointestinal	8 (4)	1 (1)	2 (1)	…	…	…	10 (5)	1 (1)
Neurologic	4 (2)	…	2 (1)	…	…	…	6 (3)	…
Renal	1 (1)	…	1 (1)	…	1 (1)	…	2 (1)	…
Obstetric/Gynecologic	2 (1)	2 (3)	…	…	…	…	2 (1)	2 (3)
Other	6 (3)	…	1 (1)	…	1 (1)	…	8 (4)	…
Death	1 (1)	…	1 (1)	…	2 (1)	…	4 (2)	…
Total with readmission or death	26 (14)	5 (7)	7 (4)	…	7 (4)	…	40 (21)	5 (7)

^a^Readmission diagnoses were not mutually exclusive and do not sum to 100%.

## DISCUSSION

Despite a number of publications documenting increased risk for severe maternal and fetal/neonatal complications from respiratory viral infections in pregnancy [1–3, 5], we report a low rate of severe adverse outcomes after index hospitalization for severe COVID-19 disease in pregnancy in our population during the first COVID wave, with 7% of pregnant participants readmitted within 18 months, no postdischarge deaths, and all readmissions occurring during the first 6 months after index hospitalization. Furthermore, we report a lower readmission rate among pregnant participants than nonpregnant participants (7% vs 21%) within 18 months of index COVID-19 hospitalization. Unfortunately, 4 (2%) nonpregnant participants died over the 18-month follow-up period, but no deaths were recorded among pregnant participants. Infection was the most common readmission diagnosis in the nonpregnant group, documented for 27 of 39 (69%). In contrast, the most common readmission diagnoses in the pregnant group were cardiac and obstetric/gynecologic, each occurring in 2 of 5 (40%). Outcomes across the 18-month period were overall better for the group who had COVID-19 in pregnancy compared to the nonpregnant group.

Although demographic and socioeconomic characteristics were similar between pregnant and nonpregnant groups in our study population, some of the difference in outcomes between groups could be explained by comorbid conditions in those hospitalized for COVID-19. Nonpregnant individuals were sicker at baseline, with a higher prevalence of diabetes, hypertension, cardiovascular and pulmonary disease, and immunocompromise. The nonpregnant group also appeared sicker during the index hospital admission and was more likely to be mechanically ventilated (84% vs 55%) and for a longer time period (13 vs 8 days) than the pregnant group. More severe COVID-19 combined with a greater prevalence of comorbidities in the nonpregnant population likely also contributed to the higher prevalence of in-hospital complications in the nonpregnant group (31% vs 17%), with pneumonia and ARDS the most commonly diagnosed complications. In both pregnant and nonpregnant populations, baseline medical comorbidities and in-hospital complications are associated with long-term adverse outcomes, including rehospitalization [6]. Thus, in our population, higher prevalence of baseline medical comorbidities and more severe COVID-19 during the index hospitalization among nonpregnant individuals may be the main drivers of post–index hospitalization readmission and death during the 18-month follow-up period. The lower rate of adverse outcomes through 18 months post–index hospitalization in the pregnant population could also be the result of less severe illness during the index hospitalization, owing to a lower threshold to hospitalize pregnant individuals at the time and pregnancy being the main coexisting condition leading to the hospitalization for COVID-19. Unlike most other comorbid conditions in the nonpregnant group, any increased risk for severe infection conferred by pregnancy likely resolved soon after delivery. Through multiple large studies including by the Centers for Disease Control and Prevention, it has been well established that SARS-CoV-2 infection in pregnancy is overall more dangerous than outside of pregnancy [7–9]. These other studies compared pregnant to nonpregnant people in the general population and found that pregnant people were more likely to experience severe COVID-19 disease. In our study, we compared outcomes between already hospitalized pregnant versus nonpregnant people and did not observe increased morbidity in pregnancy in our relatively small sample, likely as a result of differences in variable ascertainment and variety of indications for hospitalization. In our study, we made a different comparison in outcomes between pregnant and nonpregnant individuals than made by the other, larger, studies, focusing instead on 6-, 12- and 18-month outcomes among people already hospitalized. Thus, our findings do not conflict with those of earlier studies noting increased overall risk of COVID-19 in pregnant versus nonpregnant people.

Despite differences in comorbidities and COVID-19 severity between the pregnant and nonpregnant groups, the low readmission rate among pregnant participants with severe COVID-19 in pregnancy of 7% in the first 6-month period and 0% between 6 and 18 months is reassuring that pregnant individuals may not be at higher risk for long-term adverse health outcomes after COVID-19 compared to nonpregnant reproductive-aged individuals. While little is known about long-term outcomes after COVID-19 overall and in pregnancy, a high prevalence of immediate postpartum complications has been documented including 13% developing fever, hypoxemia, or readmission compared to 5% of pregnant women without COVID-19 [10]. Another published study that followed pregnant women longer-term documented higher rates of postpartum depression over the first year after COVID-19 in pregnancy [11]. However, there is currently a dearth of literature on the risk and presentation of post-COVID conditions (PCCs), future fertility and pregnancy outcomes, and future vaccine response in people who had COVID-19 in pregnancy, which future studies should address.

Strengths of our study include the relatively large cohort of 260 contemporaneously treated gender-matched individuals, including 70 pregnant people with complete baseline data and no more than 5% missingness for nonlaboratory variables. Contemporaneous examination of comparator groups is especially important in outcomes research, as prior studies have shown differences in outcomes between COVID-19 variants [12, 13]. Furthermore, outcomes may differ between individuals vaccinated against COVID-19 and those unvaccinated against COVID-19, and the contemporaneous cohorts allowed us to study an unvaccinated population in both groups. In addition, we captured rehospitalization and mortality outcomes for these participants through 18 months post–index hospitalization, a unique contribution to the current literature that addresses a gap in knowledge about the effects of severe COVID-19 in pregnancy. Because the pregnant and nonpregnant individuals were not matched on baseline characteristics, differences in baseline health and comorbidities are likely to explain some of our findings. Study weaknesses include limited generalizability to nontertiary/nonacademic hospital settings and to populations with different demographic composition. In addition, our post–index hospitalization outcomes data are not granular in nature, including broad categories of readmission diagnoses without detailed diagnostic and treatment information and limited to readmission within our own hospital system. Furthermore, the dates of index COVID-19 admission spanned >1 year, during which time thresholds for treatment and types of available and recommended treatments evolved and differed greatly between pregnant and nonpregnant individuals and between institutions. The landscape of COVID-19 infection has also changed over time, with widespread access to vaccination and changes in SARS-CoV-2 virulence such that it is not known whether our findings are applicable to patients with COVID-19 after the study period ended in 2021. We also were unable to study the impact of SARS-CoV-2 strain and immunity from vaccination or prior infection on outcomes through 18 months post–index hospitalization as vaccination and immunity data were not collected from participants. Due to the small number of pregnant individuals with COVID-19, and the small number of adverse outcomes over time, we are also unable to account for potential confounders of the relationship between pregnancy status during index hospitalization and 6-, 12-, and 18-month outcomes. It is also possible that there was differential follow-up between pregnant and nonpregnant individuals within our hospital system, biasing outcomes data if more individuals in 1 group were rehospitalized outside the hospital system than in the other group. We also did not have data on which pregnancies ended during the index hospitalization versus following hospitalization. Last, our population was enrolled before COVID-19 vaccination was available to the general public or routinely recommended in pregnancy. Future studies should investigate postacute COVID-19 outcomes, outcomes in people with COVID-19 after our study period ended in 2021, and outcomes in the context of prior immunity from infection and vaccination.

Large prospective cohort studies of pregnant and nonpregnant reproductive-aged individuals are needed to confirm the low readmission rates we report in our pregnant population. Such studies should measure risk and presentation of PCCs, relationships between COVID-19 treatments and risk of PCCs, future fertility and pregnancy outcomes, and future vaccine response in people who had COVID-19 in pregnancy. In addition, the importance of preventing adverse maternal and fetal/neonatal outcomes through vaccination pre-pregnancy or in pregnancy cannot be understated. A retrospective cohort study of 10 092 active pregnancies during the Delta wave of the pandemic found lower odds of severe or critical illness (0.08% vs 0.66%; adjusted odds ratio [aOR], 0.10 [95% confidence interval {CI}, .01–.49]) and lower odds of COVID-19 of any severity (1.1% vs 3.3%; aOR, 0.31 [95% CI, .17–.51]) among vaccinated people, and a separate study reported a significant reduction in stillbirth and preterm birth after COVID-19 vaccination in pregnancy [14], supporting universal vaccination to prevent adverse outcomes in pregnant people [15].

## CONCLUSIONS

In a large cohort of reproductive-aged people hospitalized for COVID-19 in a tertiary medical center, there was a low rate of adverse outcomes for pregnant people over the first 18 months after index hospitalization. For those with COVID-19 in pregnancy, all documented readmissions occurred within 6 months after index hospitalization; for nonpregnant individuals, readmissions decreased over time, with most admissions related to infectious diagnoses.

## Notes

***Author contributions.*** L. M. B. and A. E. W. designed the study, drafted the manuscript, and contributed to data analysis. K. E. J. contributed to data analysis. A. K., M. - R. J. and C. R. contributed to data collection and data analysis. K. J. G., I. V. B., A. A. B., A. L. C., S. Y., A. E. G., I. T. G., and K. D. contributed to study design, data collection, and dara analysis. All authors critically reviewed the manuscript.

***Patient consent.*** The study was approved by the Mass General Brigham Institutional Review Board (2020P000804), and the requirement for informed consent was waived.

***Financial support.*** K. J. G. reports funding from the NIH/National Heart, Lung, and Blood Institute (award numbers K08 HL146963 and K08 HL146963-02S1). I. V. B. reports funding from the NIH/National Institute of Allergy and Infectious Diseases (award number K24AI141036). A. A. B. reports funding from the Eunice Kennedy Shriver National Institute of Child Health and Human Development (award number K23 HD097300).
